# Mesoscopic Detection of the Influence of a Third Component on the Self-Assembly Structure of A_2_B Star Copolymer in Thin Films

**DOI:** 10.3390/polym11101636

**Published:** 2019-10-10

**Authors:** Dan Mu, Jian-Quan Li, Xing-Shun Cong, Han Zhang

**Affiliations:** 1 College of Chemistry Chemical Engineering and Materials Science, Zaozhuang University, Zaozhuang 277160, China; 2 Advanced Photonics Center, Southeast University, 2# Sipailou, Nanjing 210096, China; 3 Zaozhuang Key Laboratory of Functional Materials, Zaozhuang 277160, China; 4 Opto-Electronic Engineering College, Zaozhuang University, Zaozhuang 277160, China

**Keywords:** reverse micellar structure, copolymer, self-assembly, third component, thin film

## Abstract

The most common self-assembly structure for A_2_B copolymer is the micellar structure with B/A segments being the core/corona, which greatly limits its application range. Following the principle of structure deciding the properties, a reformation in the molecular structure of A_2_B copolymer is made by appending three segments of a third component C with the same length to the three arms, resulting (AC)_2_CB 3-miktoarm star terpolymer. A reverse micellar structure in self-assembly is expected by regulating the C length and the pairwise repulsive strength of C to A/B, aiming to enrich its application range. Keeping both A and B lengths unchanged, when the repulsion strength of C to A is much stronger than C to B, from the results of mesoscopic simulations we found, with a progressive increase in C length, (AC)_2_CB terpolymer undergoes a transition in self-assembled structures, from a cylindrical structure with B component as the core, then to a deformed lamellar structure, and finally to a cylindrical structure with A component as the core. This reverse micellar structure is formed with the assistance of appended C segments, whose length is longer than half of B length, enhancing the flexibility of three arms, and further facilitating the aggregation of A component into the core. These results prove that the addition of a third component is a rational molecular design, in conjunction with some relevant parameters, enables the manufacturing of the desired self-assembly structure while avoiding excessive changes in the involved factors.

## 1. Introduction

The chemically dissimilar linked units in block copolymers facilitate the occurrence of micro-separation, resulting in their self-assembly into rich ordered nanostructures, which have significant potential in applications such as nanowire [1], membranes [2], metamaterials [3,4], lithography [5,6], and so on. In addition to the block ratios and the interaction strength between constituting blocks, the chain architecture of block copolymers is also a well-known parameter influencing this self-assembly behavior. For example, take AB-type copolymers, whose architecture can be varied by changes in the number of repeating blocks and the chain topology, such as the simplest linear AB diblock [7], linear ABA triblock [8,9,10,11,12], AB_n_ miktoarm star [13,14,15,16,17], (AB)_n_ star [18,19,20,21], comb [22,23,24], ABAB… [25,26,27,28,29,30], (BAB)_n_ star [31,32], etc., form a wide range of structures, including body-centered cubic spheres, hexagonally-packed cylinders, bicontinuous gyroids and parallel lamellae, the perforated lamellar phase and *σ* phase. Gido’s group constructed the full phase diagram of A2B miktoarm copolymer systems, and some special morphological transformations were presented [33,34]. Recently, Li’s group examined the self-assembly of ABA_T_ block copolymers, which are composed of an AB diblock copolymer with an extra A block tethered onto the B block, using the self-consistent field theory. They found that the phase behavior depends sensitively on the topology. The local segregation of the two different kinds of blocks constrained by the chain architecture regulating the local interfacial curvature, even influencing the phase behavior, was also determined.

Theoretically speaking, increasing the number of block types can enrich the number of accessible self-assembled nanostructures, thus, an ABC triblock terpolymer, constructed by the introduction of an additional block to an AB diblock, can generate fascinating periodic structures that are not found in an AB diblock copolymer; for example, hamburger-like micelles, segmented worm-like micelles, raspberry-like micelles, double and triple helices, etc. [35,36,37,38,39]. However, owing to the rapid expansion of the parameter space, the increase in the number of block types brings great challenges to structure searching and control [40]. A further illustration starts from the simplest linear AB diblock copolymer, which is modeled via two parameters (i.e., χ_AB_*N* and *f*_A_, where χ_AB_ is the Flory–Huggins interaction parameter, *N* is the chain length, and *f*_A_ is the chain length ratio of A component). When one additional block type is introduced, an ABC triblock copolymer is constructed, and at least five parameters are needed, i.e., χ_AB_*N*, χ_BC_*N*, χ_AC_*N*, *f*_A_, and *f*_B_. If the number of block types increases further, the number of parameters to describe the model will be dramatically expanded to the extent that is hard to bear.

In our previous work, two types of A_n_B miktoarm star copolymers were studied—one was an H-shaped structure [41], and the other was a Y-shaped structure [42]. Both copolymer thin film systems were self-assembled into a hexagonal cylindrical arrangement; especially, the extra A arm in the chain architecture makes the phase behavior of the H-shaped copolymer different from that of the Y-shaped copolymer. Compared with the Y-shaped copolymer thin film, the regulation of the cylindrical size in the H-shaped copolymer thin film is completed with the aid of vacancies; thus, the resultant size distribution is wider. However, the self-assembled cylinders are all composed of B component as the core, which limits its function. In view of the new structure implying new functions and properties, the search for the formation conditions of the reverse micelle is essential and is beneficial to expand the potential function via the reformation of the chain architecture. Inspired by the increase in the block types prompting more abundant self-assembled structures, the according growth in the number of descriptive parameters is also taken into account, and a third component is introduced into each arm of a Y-shaped copolymer, generating a resultant (AC)_2_CB 3-miktoarm star terpolymer. Furthermore, the corresponding self-assembly mechanism of the reverse micelle is also illustrated.

## 2. Models and Parameter Settings

Mesoscopic modeling is a flexible and powerful theoretical tool to study inhomogeneous polymeric systems. Among the various modeling methods, MesoDyn has been validated as an effective approach to explore the phase behavior of polymer systems at the mesoscale [43,44,45,46,47,48]. Furthermore, MesoDyn facilitates treating various chain architectures of polymers, from linear [49] to branched [41,42,50]; MesoDyn is a mature method to investigate the phase behavior of flexible block copolymers.

The detailed derivation and theory of MesoDyn is readily found in several reports, so only a brief description is given here. The intrinsic theory of the MesoDyn method is based on the dynamic mean-field theory developed by Fraaije [51,52]. Here, the phase separation dynamics and ordering processes of the polymeric systems are described by Langevin equations, and the Gaussian chain density functionals are established between the external potential fields and the density fields for each bead type by a one-to-one correspondence. Moreover, the intrinsic chemical potentials are the functionals of the external potentials and density fields. Hence, the coupled Langevin equations are capable of describing the relationship between the time derivatives and the intrinsic chemical potentials. As a result of the association between the noise source and the exchange kinetics coefficients, a closed set is formed to be efficiently integrated into a cubic mesh for the iteration of the inner loop by a Crank–Nicholson scheme [53]. When all beads are treated with the same size for simplicity, the chain topology primarily depends on the coarse-graining mode.

**Scheme 1:**
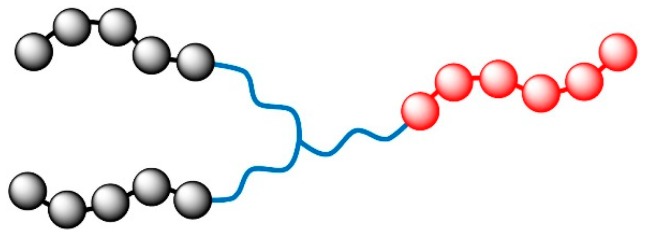
Schematic representation of the (AC)_2_CB 3-miktoarm star terpolymer. The black and red spheres denote the A and B beads, respectively. The three blue lines denote the inserted C segments of the same length, each consisting of an increasing number of C beads.

In consideration of keeping the main frame of polymer unchanged, the topological structure of the A_2_B 3-miktoarm star is preserved, and three segments consisting of a third component (C) are inserted along the three arms from the junction point, forming an (AC)_2_CB 3-miktoarm star terpolymer composed of two copolymer arms of AC and one copolymer arm of CB, whose schematic structure is shown in Scheme 1. Furthermore, the numbers of A and B beads in each segment are also reserved as 5 and 6, respectively, and the interaction parameter between A and B beads is set as 4.68 kJ·mol^-1^; therefore, both are kept the same as that in our previous work [42]. The three appended C segments are the same length, and the number of C beads in each C segment is progressively increased from 1 to 6, resulting in six individual systems, named as 3C1, 3C2, 3C3, 3C4, 3C5, and 3C6 systems (additionally, the name for the system without C segment is C0). Apart from the chain length of the C segments, the interactions of C beads with A and B beads are also examined. Thus, four pairwise interactions are taken into account, i.e., *E*_CA_ = 0 kJ·mol^−1^ and *E*_CB_ = 0 kJ·mol^−1^, *E*_CA_ = 5 kJ·mol^−1^ and *E*_CB_ = 5 kJ·mol^−1^, *E*_CA_ = 1 kJ·mol^−1^ and *E*_CB_ = 5 kJ·mol^−1^, and *E*_CA_ = 5 kJ·mol^−1^ and *E*_CB_ = 1 kJ·mol^−1^, which are referred to as C[00], C[55], C[15], and C[51], respectively, and all the interaction parameters are listed in Table 1. From the comparison of C beads with A and B beads, the former two belong to non-preferential interactions, i.e., there is no preference for the C bead to interact with either A or B bead; whereas, the latter two belong to preferential interactions, where the affinity of the C bead lies with A beads and B beads for C[15] and C[51], respectively.

Compared with three dimensions (3D), some structures that result from the simulation in two dimensions (2D) may be missed, such as bi- or tri-continuous gyroid structures. However, in industry, many materials, benefiting from 2D ordered structures fabricated in thin films, manifest great potential, which reveals the structure control in 2D space possesses an essential significance that cannot be replaced. Therefore, all the simulations in this paper are carried out in the *L*_x_ × *L*_y_ × *L*_z_ spatial lattice of a 50 × 50 × 5 nm^3^ grid, whose width in the Z axis is 10% of the width in either the X or Y axis, to model thin films. Additionally, the bond length is set as 1.1543 to ensure the isotropy of all grid-restricted operators. To neglect the size influence on self-assembly, all beads are an equal size of 0.01 nm^3^ for simplicity. In MesoDyn simulations, the bead diffusion coefficient provides the link between the real timescale of the simulation and the dimensionless units, and a default value of 1.0 × 10^-7^ cm^2^·s^-1^ is adopted. Owing to the inverse relationship of the dimensionless noise parameter to the level of thermal noise applied, a maximum value of 100 for the noise-scaling parameter is employed to realize the minimum in noise. Lastly, the compressibility parameter is fixed at 10.0, the dimensionless time step is set as 0.5, and each simulation is carried out at a constant temperature of 400 K for 1.0 × 10^5^ steps (*i.e.*, 5 ms).

## 3. Results and Discussion

**Figure 1:**
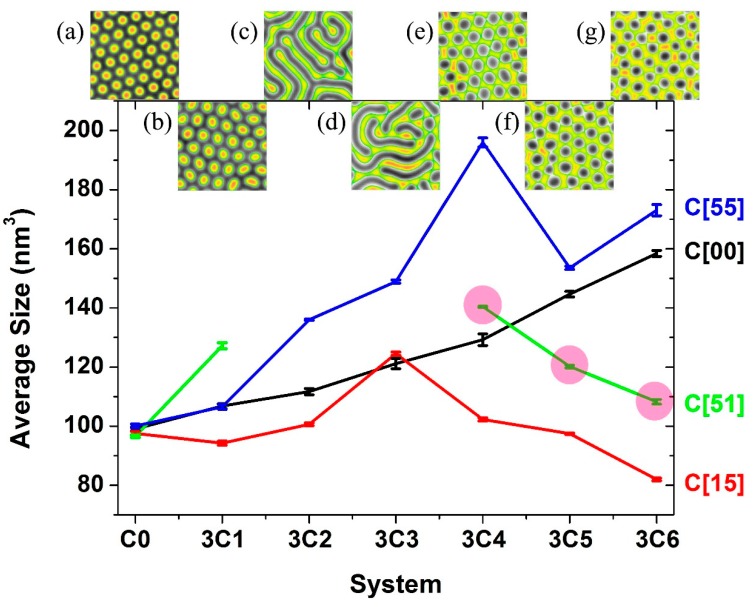
Average sizes of different systems forming the cylindrical structure. The black, blue, red, and green lines correspond to the interactions of C[00], C[55], C[15], and C[51], respectively. The two missing points on the green line are the systems assembled into non-cylindrical structures. The three pink circle-marked points are the systems assembled in the reverse micellar structure. The subfigures (**a**)–(**g**) are the density slices at the final equilibrium state along the X–Y plane for the systems from C0 to 3C6 under C[51] interaction, respectively.

Most of systems assemble into cylindrical structures, whose average sizes are readily calculated and are shown in Figure 1. For a clear comparison of the reformation of the molecular structure, the systems with only one C bead as the joint point in the A_2_B topology are also simulated under the interaction situation of C[00], C[55], C[15], and C[51]. The relatively low composition of C beads compared with that of A and B beads, as well as the innermost location of the C bead, both severely restrict the C bead’s influence on the other components, resulting in no apparent difference in the average sizes under various interaction conditions. With the increase in the length of the C segment, the tendencies in the change of the average size under various conditions present evident differences. Only the average sizes of systems with the C[00] interaction show an almost direct proportional change tendency with the increasing length of each C segment. For the same kind of non-preferential interaction of C[55], whose change tendency for the average size is similar to that of C[00], the strong repulsion of C beads to both A and B beads results in a larger average size than that of C[00]. Particularly, the 3C4 system exhibits the highest value of the average size and breaks the monotonically increasing tendency of average size, which will be given a detailed analysis later. With increasing C beads, the change tendency for the average sizes with C[15] interaction approximately rises at first and then decreases. Except for the 3C3 system, whose average size is the highest and higher than the same system with the C[00] interaction, the other systems with the C[15] interaction all possess an average size that is less than that of the corresponding system with the C[00] interaction. The systems with the C[51] interaction are relatively complicated, as only the single addition of a C bead per C segment results in a tremendous increase in the average size of the 3C1 system. Then, two non-cylindrical structures are formed for 3C2 and 3C3 systems. Finally, the 3C4, 3C5 and 3C6 systems are cylindrical in structure, but the constituent components of the core and shell are reversed, and a decreasing trend for their average sizes is displayed.

**Figure 2:**
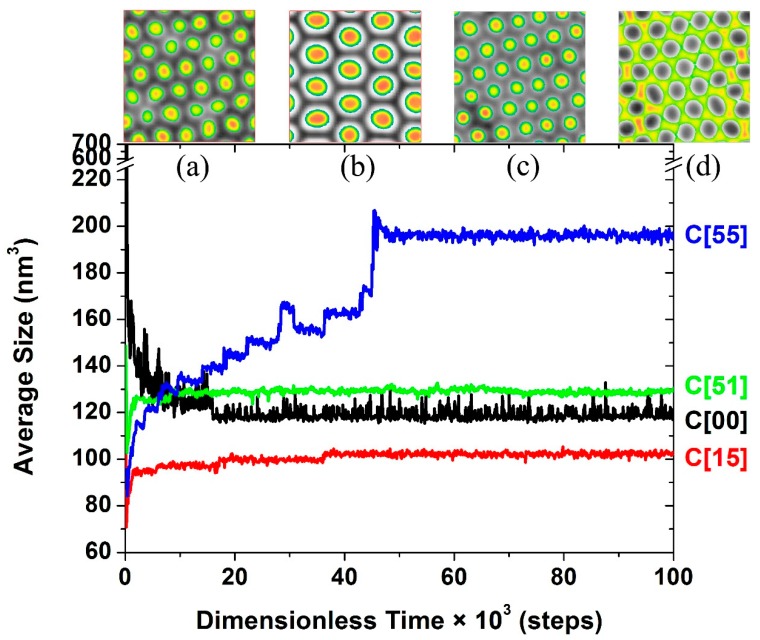
Change in average size for the same 3C4 system under C[00], C[55], C[15], and C[51] interaction conditions are shown in black, blue, red, and green lines, respectively, whose density slices at the final equilibrium state along the X–Y plane are shown in subfigures (**a**)–(**d**), respectively.

Taking the 3C4 system as an example, from a track of average size with time shown in Figure 2, different tendencies under various interaction conditions are found. Clearly different from the trend of the other lines, C[00] presents a decreasing trend, which reveals the splitting mechanism of the assembled micelles. Moreover, the non-preference, as well as an even lack of interaction, of C beads and A and B beads, and the innermost location of the C component enlarging the distance between A and B component both result in a weakening interaction between the A and B components, making the formation of regular micelles unstable. Therefore, the largest amplitude of variation for C[00] can be interpreted. Both the curves of C[15] and C[55] exhibit a stepwise-increasing trend, indicating the fusion mechanism of the assembled micelles. Compared to the C[15] interaction, the non-preferential but strong repulsion between C beads to both A and B beads under the C[55] interaction intensifies the competition for forming a core and shell composition, which needs more steps in the fusing of small micelles into large micelles, costing more time for the adjustment process. A reverse micellar structure is generated under the C[51] interaction. The stronger repulsion between C and A beads immediately facilitates the initial formation of the core, so there is nearly no evident fusing process responsible for converting small micelles into large micelles. Additionally, the weaker repulsion between C beads to B beads is helpful in stabilizing the assembled micelles, resulting in the smallest amplitude of variation in average size.

### 3.1. Self-Assembly under Non-Preferential Interactions

**Figure 3:**
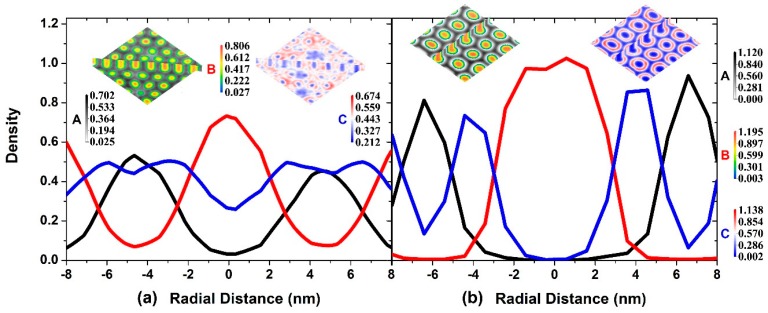
For 3C4 systems at the final equilibrium state, whose density profiles of A, B, and C beads of randomly selected individual cylinders under (**a**) the C[00] interaction and (**b**) C[55] interaction, starting from the center point of the cylinder (set as the original point) and moving along the radial direction, and denoted in black, red, and blue lines, respectively. Accordingly, two density images are inserted and whose color maps of A, B, and C beads are in white–black, reverse rainbow, and blue–white–red, respectively. Each density image consists of two density slices; one density slice is along the X–Y plane, and the other density slice goes through the cylindrical axis along one cylindrical array, with the latter one being vertical to the former one.

The C[00] and C[55] interactions are two typical representatives of non-preferential interactions, but their distinctly different repulsive strengths for the interaction between C beads and A and B beads cause their different self-assembly structures, as shown in Figure 3. For the C[00] interaction, a lack of interaction between C beads and both A and B beads does not give rise to influence over B beads in the core and A beads in the corona, or to influence over the wide distribution of C beads with a relatively high density. Conversely, for the C[55] interaction, not only does a squeezing effect generated from the strong repulsion between C beads to both A and B beads favor the concentration of an A bead-aggregating zone and B bead-aggregating zone, respectively, but also the steric hindrance from C beads located between A and B beads contributes to producing a wide range of core regions; consequently, the final configuration is equivalent to a vesicle structure, if one does not take component C into account. A detailed illustration of the fusing mechanism of the self-assembly is made in the Figure S1–S3 of Supplementary Materials part via three representative analyses of the density distribution.

### 3.2. Two Transitional Systems

**Figure 4:**
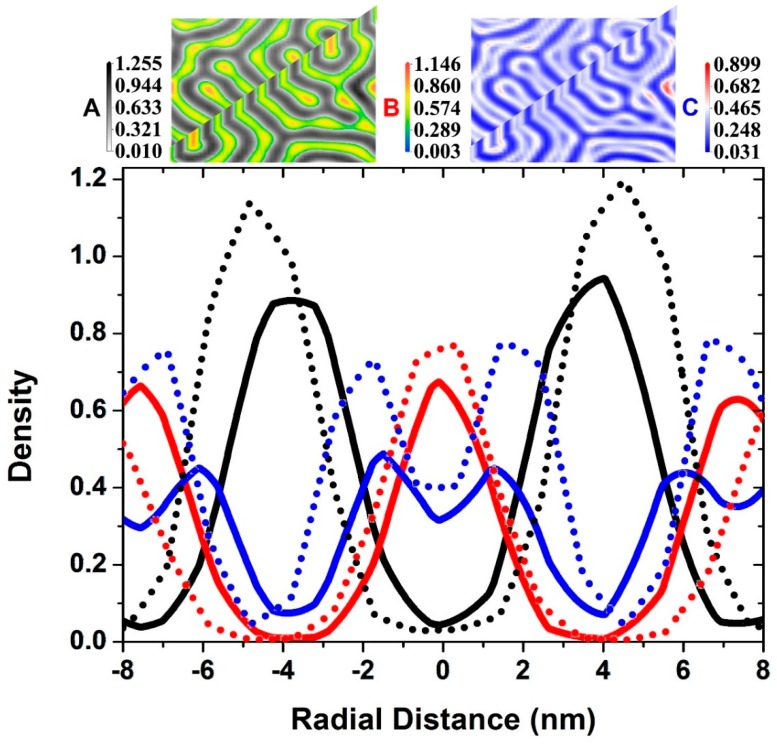
The density profiles of A, B, and C beads of another three adjacent aggregates consisting of B beads are denoted in black, red, and blue lines, respectively, for C[51] interactions at the final equilibrium state. The solid and dotted lines correspond to the 3C2 and 3C3 systems, respectively. Two density images selected from the 3C2 system of A–B beads (left) and C beads (right) are shown on the top and consist of two density slices, with one density slice along the X–Y plane and the other density slice aligned vertically across parallel lamellae (the latter one is vertical to the former one).

As shown in Figure 1, three different structures are produced with increasing numbers of C beads, i.e., the cylindrical structure with B beads constituting the core part (C0 and 3C1 systems), deformed lamellar structure (3C2 and 3C3 systems), and cylindrical structure with A beads constituting the core part (3C4, 3C5, and 3C6 systems) for C[51] interactions. Thus, the two intermediate systems, 3C2 and 3C3, are regarded as the transition from the regular cylindrical micellar structure to the reverse cylindrical micellar structure, whose density profiles are shown in Figure 4. The same change tendency occurs for the same constituting beads in the 3C2 and 3C3 systems, indicating the same kind of assembled structure. However, compared with the 3C2 system, the 3C3 system presents two distinct differences: One is the relatively larger distance between the same kinds of aggregates, and the other is the relatively higher peak value in the density profiles consisting of the same component, which can be understood from the steric hindrance and strong repulsion from C beads. Due to the location of the C beads accumulating between adjacent A aggregates and B aggregates, the increase in C beads of the 3C3 system leads to more occupation of C aggregates. The strong repulsion from more C beads, resulting in both A aggregates and B aggregates being ‘pushed’ far away from C aggregates, presents as relatively larger distances between the same kind of aggregates. Moreover, every A aggregate or B aggregate is sandwiched in the middle by two C aggregates, with the increasing repulsion resulting from the additional C beads enhancing the ‘squeezing effect’, causing the A beads or B beads to be ‘squeezed’ inward, and this action represents the relatively higher peak value of the density profiles consisting of the same component. Different from the regular cylindrical structure with the B beads as the definite core part and the A/C beads surrounding, there is no definition for the core or shell in the deformed lamellar structure, only the duplication of A aggregates to C aggregates to B aggregates to C aggregates can be determined. Therefore, as an inference, the possibility of forming the reverse cylindrical micellar structure when further C beads are added to the inserted C segments, advancing the flexibility of both A and B segments, can be reasonably introduced.

### 3.3. The Self-Assembly of the Reverse Cylindrical Micellar Structure

**Figure 5:**
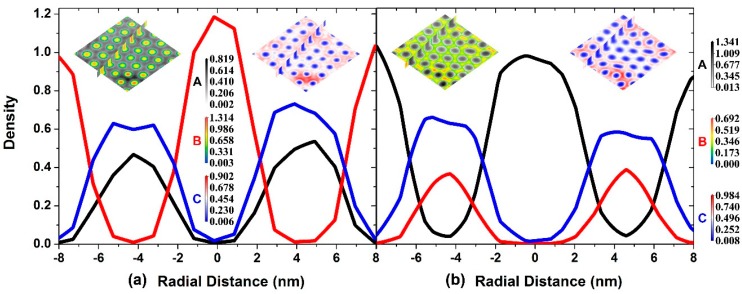
Density profiles of the 3C4 system at the final equilibrium state for (**a**) C[15] interaction and (**b**) C[51] interaction, and the densities of A, B, and C beads are denoted in black, red, and blue lines, respectively. In each subfigure, two density images are inserted. The images represent A–B beads (left) and C beads (right), consisting of two density slices with one density slice along the X–Y plane and the other density slice intersecting the cylindrical axes along one cylindrical array (the latter one is vertical to the former one).

For comparison, the same kind of density profiles for the 3C4 system affected by the C[15] interaction are shown in Figure 5a, whereby the interaction between C beads and B beads is significantly stronger than that between C beads and A beads. In this case, the C aggregates are dramatically separated from the B aggregates, whereas the C aggregates share the same location with the A aggregates, and both play the role of the shell. As predicted, when the inserted number of C beads in each C segment grows to four or even more, the resultant systems self-assemble into the reverse cylindrical micellar structure under the C[51] interaction, and the density profiles of the 3C4 system affected by the C[51] interaction are shown in Figure 5b. Resulting from the flexibility of the C segments being enhanced, which facilitates the A beads aggregating as the core part, as well as the stronger repulsion of C beads to A beads than to B beads, the separation of the A-aggregating peak and the C-aggregating peak occurs; thus, both of A- and B-aggregating play the role of the shell.

Different from the rising trend (or roughly rising trend) in the average size changing with the increase in the number of inserted C beads present in non-preferential interaction systems (i.e., C[00] and C[55]), a decreasing trend is present in the systems with four or more C beads in each inserted C segment under a preferential interaction (i.e., C[15] and C[51]), which results from their distinguishing structures. At first, leaving aside the position exchange of the A and B components under two preferential interactions, an outstanding feature lies in the overlapped distribution of the C beads and the A or B beads also making up the shell part and is distinct from peaks associated with the aggregation of C beads between the aggregation of A beads and the aggregation of B beads under a non-preferential interaction. Furthermore, the wide distribution of C beads and the sharing scope of A or B beads reinforce the repulsive strength interacting with the components making up the core. Thus, the increasing repulsion from more C beads ‘squeezes’ the components of cores inward to become more condensed, resulting in the production of smaller-sized cylinders; this result is presented as the decreasing average sizes in Figure 1.

**Figure 6:**
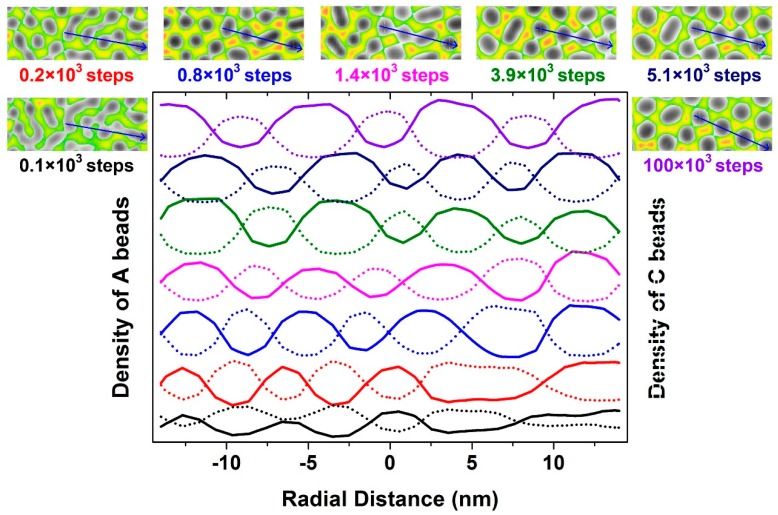
Density distributions of A beads (solid line) and C beads (dotted line) along the selected route—labeled in blue with arrows in the density slices at different times, i.e., 0.1 × 10^3^ steps (in black), 0.2 × 10^3^ steps (in red), 0.8 × 10^3^ steps (in blue), 1.4 × 10^3^ steps (in magenta), 3.9 × 10^3^ steps (in olive), 5.1 × 10^3^ steps (in navy), and 100× 10^3^ steps (in violet). The density slices are displayed at the top and from left to right, according to the time sequence.

Due to the particular structure formed by the 3C4 system under the C[51] interaction, the formation process of the reverse cylindrical micellar structure is necessarily studied, as shown in Figure 6. The repulsion of C beads and A beads is stronger than that of the former and B beads; thus, no matter the time, the changing trend of A- and C-bead densities are converse, i.e., when the density of A beads goes up, the density of C beads must go down. At 0.1 × 10^3^ steps, an initial partition forms via the aggregation of the same component, with four peaks of A beads found following the selected route. Additionally, not only are the aggregates in various patterns, but also the value and scope of the four peaks are not distinct. After 0.1 × 10^3^ steps, further aggregating of the same component causes the growth in densities of the four peaks. In the following 1.2 × 10^3^ steps, along with the fusion of small aggregates and the splitting of large aggregates happening simultaneously, the irregularity of aggregating patterns is reduced, and the surrounding C-shells are gradually constructed. The completion of normal-shaped cylinders along the selected route is first gained in 2.5 × 10^3^ steps, but the acquirement of a uniform density distribution among the four cylinders is achieved in the subsequent 1.2 × 10^3^ steps. Finally, including the four cylinders mentioned above, other cylinders also achieve the normal shape, even layout of position and uniform density distribution. Three long-chained C segments situated between A and B segments are helpful for inverting the core-forming component, i.e., A beads aggregating in the core region, in conjunction with the concurrent fusion and splitting among aggregates, brings about the least time needed to reach the equilibrium state of average size, whereby the amplitude of variation in the changing curve of the average size is also the smallest, as shown in Figure 2.

## 4. Conclusions

The self-assembled structures and mechanism of an (AC)_2_CB 3-miktoarm star terpolymer in a thin film have been studied via MesoDyn simulations. To minimize the number of factors influencing the self-assembly, only the length of C segment and the interaction between the C component and A/B component are changed.

Owing to the extraordinary high value of the average size of the 3C4 system affected by C[55], as well as the 3C4 system being also on two decreasing lines for the average size with the increase in C segment affected by C[15] and C[51], the 3C4 system is selected as a representative system for further analysis. Different forming processes can be speculated upon from the trajectory of the average size with time under different interaction conditions, that is, a splitting process (under the C[00] interaction), multistep coalescence process (under the C[55] interaction), three-step coalescence process (under the C[15] interaction), and speedy uncertain process with no clear change (under the C[51] interaction).

After a detailed analysis of the densities, the associated mechanisms can be revealed definitely. For the C[00] interaction, where the repulsions between C beads and A and B beads are both zero, leading to a nonselective distribution of C beads in A- and B-aggregating regions, the interaction between A and B beads weakens, and then, the unstable cylindrical structure shows a wide-amplitude of change in the average size starting from the splitting of several bulk aggregates. For the C[55] interaction, where the repulsions between C beads and A and B beads are equal and both strong, giving rise to a C shell forming between A and B aggregations, the coalescence of pairwise smaller aggregates is accompanied by the change in the surrounding type of C shell and is presented as a multistep coalescence mechanism for the self-assembly process. For the C[15] or C[51] interaction, where the C beads preferentially repulse B beads or A beads, causing the covering of C-aggregating regions on A-aggregating regions or B-aggregating regions, respectively, to compose an outer shell. This ‘squeezing effect’ from such a heterogeneous outer shell strengthens with the C-segment length, so a decreasing trend in average size can be interpreted. The most delightful thing is the appearance of the reverse cylindrical micellar structure, and it is also a result of rational molecular design, which happens in the 3C4 system under the C[51] interaction. This result fills in the shortage of such reverse micellar structures by changing only two influential factors and offers a significant opportunity in the design of materials with novel structure, which enables the fabrication of a wide range of useful devices.

## Figures and Tables

**Table 1:** Input parameters (*E*_ij_) information.

*Eij* (kJ·mol^−1^)	A	B	C[00]	C[55]	C[15]	C[51]
A	0	4.68	0	5	1	5
B	4.68	0	0	5	5	1
C[00]	0	0	0	-	-	-
C[55]	5	5	-	0	-	-
C[15]	1	5	-	-	0	-
C[51]	5	1	-	-	-	0

## Supplementary Materials

The following are available online at https://www.mdpi.com/2073-4360/11/10/1636/s1, Figure S1: For 3C4 systems, whose density profiles for the B and C beads of two adjacent cylinders at different simulation steps, where black, red, blue, magenta, olive and green lines represent the steps numbered 0.2 × 10^3^, 0.3 × 10^3^, 2.0 × 10^3^, 3.6 × 10^3^, 5.0 × 10^3^ and 5.6 × 10^3^ steps, respectively for C[55] interactions, and the according snapshots of the density images are also displayed on the top. The original point corresponds to the position between the two cores of the adjacent cylinders. Figure S2: For 3C4 systems, whose density profiles of the B and C beads of two adjacent cylinders are provided at different simulation steps, where black, red, blue, magenta, olive and green lines represent the steps numbered 3.3 × 10^3^, 4.3 × 10^3^, 12.1 × 10^3^, 14.5 × 10^3^, 15.5 × 10^3^ and 17.4 × 10^3^ steps, respectively for C[55] interactions, and the corresponding snapshots of the density images are also displayed on the top. The original point corresponds to the position between the two cores of adjacent cylinders. Figure S3:For 3C4 systems, whose density profiles of B and C beads of two adjacent cylinders are provided at different simulation steps, where black, red, blue, magenta, olive and green lines represent the steps numbered 17.4 × 10^3^, 18.8 × 10^3^, 31.7 × 10^3^, 41.7 × 10^3^, 47.0 × 10^3^ and 48.0 × 10^3^ steps, respectively, and the corresponding snapshots of the density images are also displayed on the top. The original point corresponds to the position between the two cores of adjacent cylinders.
