# Supplementary material for: Mesoscopic Detection of the Influence of a Third Component on the Self-Assembly Structure of A2B Star Copolymer in Thin Films
Source: Polymers (Basel). 2019 Oct 10;11(10):1636. doi: 10.3390/polym11101636 (PMC6835291; doi:10.3390/polym11101636)
Supplement: Supplementary file 1 [file polymers-11-01636-s001.pdf]

Supplement

Here, we take 3C4 system under the C[55] interaction as an example to elaborate the fusing mechanism during self-assembly, the change of density distributions along the radial direction of two adjacent cylinders at several typical times are used to describe the fusing process quantificationally. In Figure S1, at the very beginning of 0.2×10^3 steps, owing to the strong repulsion between C beads and A and B beads, an embryonic form of two cores can be identified from two broad peaks of B beads. In the next 0.1×10^3 steps, the initial aggregating of C beads between two cylinders exerts the squeezing effect, which accelerates the aggregation of B beads, resulting in the rapid growth of the B-bead density. Subsequently, the synchronous increases in the C-bead density explicitly promotes two separate cylinders. The expelled B beads from the region between the two peaks are collected into two peaks of B beads, leading to two broad-range peaks. With the gradual approach of the two cylinders in the next 4.7×10^3 steps, the C beads between them are accordingly extruded, which is represented as a gradual decrease in the density of C beads. Up to 5.6×10^3 steps, the coalescence of the two cylinders is completed, as confirmed from the large and similar densities of B beads with wide-ranges covering the scope of the two original cylinders, as well as the nearly zero-value density of C beads in the newly born bigger cylinder.

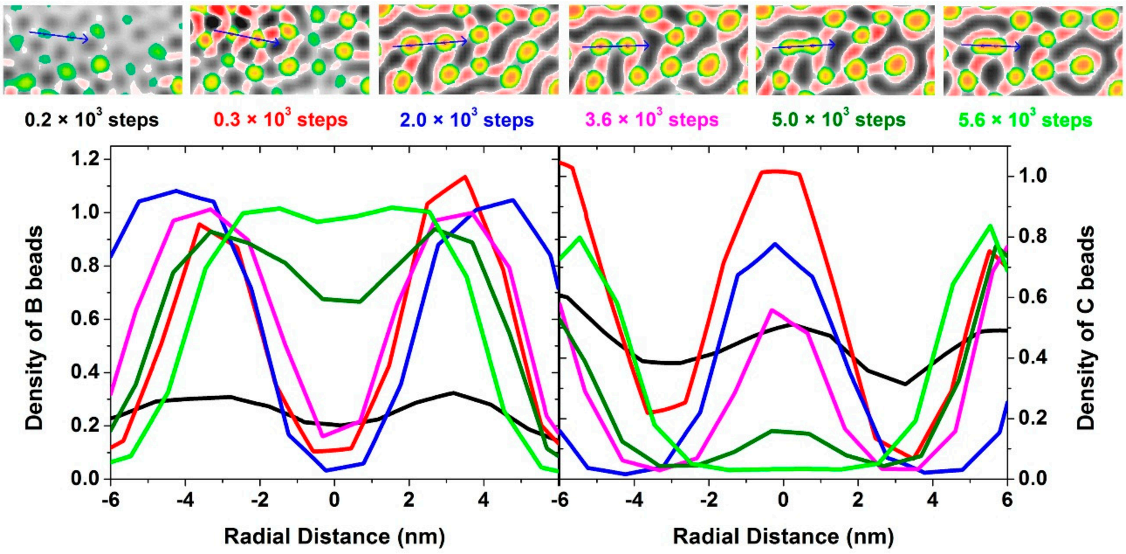

Figure S1. For 3C4 systems, whose density profiles for the B and C beads of two adjacent cylinders at different simulation steps, where black, red, blue, magenta, olive and green lines represent the steps numbered 0.2×10^3, 0.3×10^3, 2.0×10^3, 3.6×10^3, 5.0×10^3 and 5.6×10^3 steps, respectively for C[55] interactions, and the according snapshots of the density images are also displayed on the top. The original point corresponds to the position between the two cores of the adjacent cylinders.

Similarly, coalescence of two smaller cylinders into one bigger cylinder, whose formation process is described by the analysis of the densities of B and C beads, can be found nearby, as shown in Figure S2. At 3.3×10^3 steps, the initial structures of the two smaller cylinders are the same and consist of B beads aggregating as the core and C beads surrounding the core to be shell-like. In the next 1.0×10^3 steps, as the two cylinders approach each other, their surrounding C shells also approach each other. Suffering from the approaching strong repulsion from the opposite core, the constituting C beads in the inner shell are repelled to the outer shell, resulting in a decrease in the density of the inner shell and an increase in the density of the outer shell. As the two cylinders substantially approach in the following 7.8×10^3 steps, an unequal distribution in their outer shells is induced via a delivery of a certain amount of C beads through the gap between two cylinders. With

the distance between the two cylinders becoming shorter and shorter, the two cores progressively fuse into one with the B beads filling the gap between the two cylinders. Repelled from the B beads and their steric hindrance, the transfer quantity of C beads through the gap becomes less and less until it is zero; this action costs 3.4 × 10³ steps. In the following 1.9 × 10³ steps, the resultant larger cylinder with an oval intersecting surface adjusts the distribution of both the core composed of B beads and the shell composed of C beads to be a regular circle intersecting surface, which continues until both the B beads and the C beads distribute evenly at the same radial position.

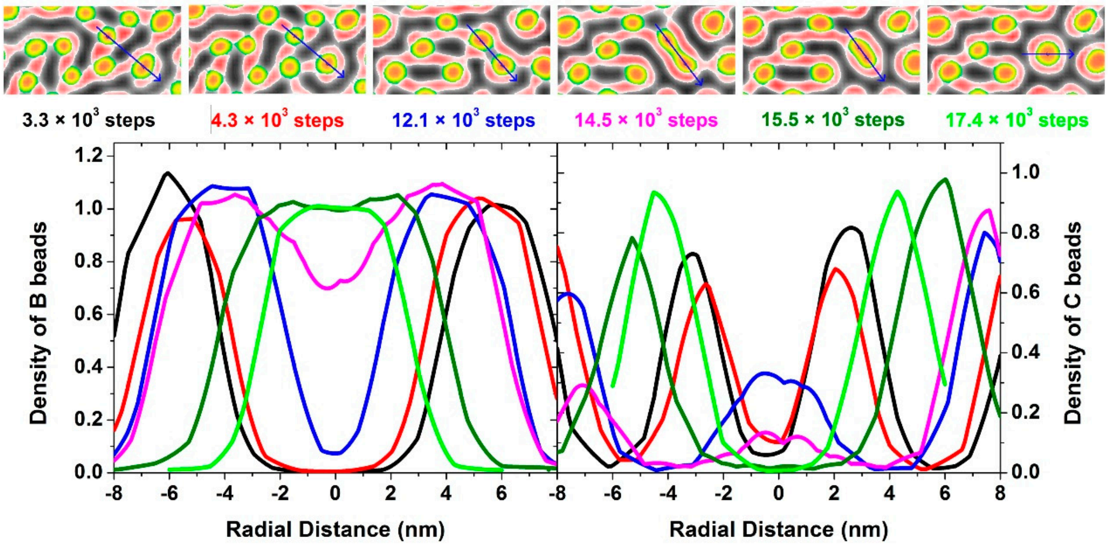

Figure S2. For 3C4 systems, whose density profiles of the B and C beads of two adjacent cylinders are provided at different simulation steps, where black, red, blue, magenta, olive and green lines represent the steps numbered 3.3 × 10³, 4.3 × 10³, 12.1 × 10³, 14.5 × 10³, 15.5 × 10³ and 17.4 × 10³ steps, respectively for C[55] interactions, and the corresponding snapshots of the density images are also displayed on the top. The original point corresponds to the position between the two cores of adjacent cylinders.

After the splitting and coalescence of the core parts of each individual cylinder, the transfers of stable surrounding C shells occurs, as shown in Figure S3. The illustration starts from six cylinders in the last snapshot in Figure S2 at 17.4 × 10³ steps, *i.e.*, two isolated cylinders numbered 2 and 6, as well as four cylinders connected by shared C shells numbered 3, 1, 5 and 4. To reach a lower free energy, more ordered structures are needed, as only two ordered cylinders (*i.e.*, Cylinder 2 and Cylinder 6) with quasi-surrounding C shells are not enough. Thus, in 1.4 × 10³ steps, both of Cylinder 2 and Cylinder 6 open their C shells to connect with Cylinder 4 and Cylinder 6, respectively. In the next 12.9 × 10³ steps, two additional connections from the C shells of both Cylinder 2 and Cylinder 6 to Cylinder 3 are built to form a wide range of connections among the six cylinders, building up a channel to transfer the constituents among different cylinders. After 10.0 × 10³ steps, the first stagewise redistribution of components among the six cylinders is completed by getting rid of Cylinder 6 and Cylinder 5 from the former large-scale association; additionally, the two opposite-part C shells of Cylinder 1 and Cylinder 2 are gradually formed via the filling of C-shortage areas with C beads from the newly born association of Cylinder 1, Cylinder 2, Cylinder 3 and Cylinder 4. Simultaneously, the complements of the C shells of Cylinder 3 and Cylinder 4 are completed following a similar transferring process of C beads. With the formation of intact C shells, the distances between adjacent cylinders are increasing under the strong repulsion from C beads. Subsequently, all connections consisting of C beads among cylinders are broken off, leaving independent cylindrical micelles surrounded by respective C-shells with uniformly distributed C beads. During the remaining time of 52.0 × 10³ steps, both the arrangement of cylinders and the structure of individual cylinder keep unchanged essentially as it at 48.0 × 10³ steps.

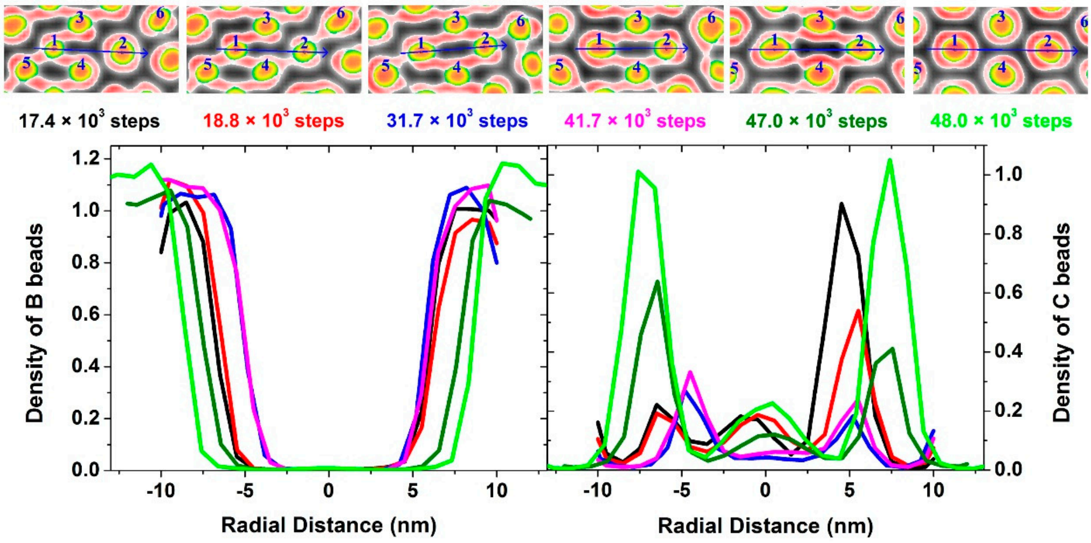

Figure S3. For 3C4 systems, whose density profiles of B and C beads of two adjacent cylinders are provided at different simulation steps, where black, red, blue, magenta, olive and green lines represent the steps numbered 17.4 × 10^3, 18.8 × 10^3, 31.7 × 10^3, 41.7 × 10^3, 47.0 × 10^3 and 48.0 × 10^3 steps, respectively, and the corresponding snapshots of the density images are also displayed on the top. The original point corresponds to the position between the two cores of adjacent cylinders.
